# Preliminary Behavioural Observations of Horseback Safaris: Initial Insights into the Welfare Implications for Horses and Herbivorous Plains Game Species

**DOI:** 10.3390/ani12040441

**Published:** 2022-02-11

**Authors:** Evelyn Hodgson, Nicola J. Rooney, Jo Hockenhull

**Affiliations:** Bristol Veterinary School, University of Bristol, Langford House, Bristol BS40 5DU, UK; nicola.rooney@bristol.ac.uk (N.J.R.); jo.hockenhull@bristol.ac.uk (J.H.)

**Keywords:** animal welfare, equestrian tourism, game species, horse, response behaviour, safari

## Abstract

**Simple Summary:**

Horseback safari rides, where tourists are led by experienced guides on horseback to find and observe wildlife, are a popular activity in Africa. However, close encounters between horses and wildlife on safari rides may be stressful for both types of animals. In this study we looked at the behaviour of horses and wildlife during close encounters on horseback safari rides, focusing on their behaviour at the start and end of each encounter, and the most extreme behaviour seen. Encounters with seven wildlife species were observed, all large herbivores. The seven species differed in their behaviour towards the horses. The horses also differed in their behaviour towards the different wildlife species, being more wary of giraffe. Horses generally approached the wildlife species at walk and few flight behaviours were observed. Further studies, including those incorporating physiological measures of stress, are recommended.

**Abstract:**

In Africa, wildlife-watching experiences create substantial revenue from tourists that can finance wildlife conservation. Horseback safaris, where an experienced guide takes guests through the bush on horseback to observe plains game species, are a popular activity. Close encounters between ridden horses and game species are unnatural and potentially stressful situations, and horseback safaris may have adverse impacts on both the horses and the wildlife they have come to observe. This study aims to provide a preliminary insight into the behavioural responses of horses and herbivorous plains game species, including giraffe, zebra and impala, as a proxy measure of the potential welfare implications of horseback safaris. Seventeen group safari rides were observed encompassing 72 encounters with plains game species. Game species differed in their response to encounters with the horseback safari ride. Equine response behaviour appeared to be influenced by the species of game encountered. Horses seemed more wary of giraffe than other species, with a higher percentage of horses showing stationary and retreat behaviour at the start of giraffe encounters. They were also most likely to shy at giraffe. The behavioural responses suggest that game encounters can elicit a stress response in both animal groups, although it is not usually extreme, potentially indicating that some degree of habituation has occurred. Balancing the welfare of both the horses and the plains game species along with tourist preferences may be challenging in this context.

## 1. Introduction

In Africa, the financial cost of wildlife conservation is significantly underpinned by income from tourists [1]. Safari adventures are a major part of this, and there are a plethora of wildlife watching experiences available, from luxury hot air balloon rides to guided off-road drives in 4 × 4 s and horseback safaris. The direct and indirect impact of wildlife tourism on the resident wildlife can be considerable [2], but is typically balanced against the potential benefits such as raised public awareness of conservation issues and the generation of revenue to finance conservation work [3,4,5]. Tourist activity can damage natural ecosystems [6] and has been associated with an increase in physiological indicators of stress in a range of species, including elephants [7], gorillas [4], howler monkeys (*Alouatta pigra*) [8], and capercaillie (*Tetrao urogallus*) [9], often with corresponding changes to their behaviour, e.g., in mountain hares (*Lepus timidus*) [10] and elephants (*Elephas maximus*) [11].

Horseback safari rides are a popular activity in Africa, with a range of different lengths and difficulties available to suit tourist requirements. During these rides, an experienced guide takes the guests through the bush on horseback, to find a range of plains game species. The term “plains game” is commonly used in the safari industry (Cumming 1989) to refer to herbivorous plains dwelling species, including zebra (*Equus quagga*) and species of antelope. Safari rides typically focus their encounters on plains game species which are herbivorous, not predators. These are more suitable for close tourist encounters on horseback, particularly for beginner riders, than the so-called dangerous game species such as the ‘Big Five of Africa’ (lion (*Panthera leo*), leopard (*Panthera pardus*), rhinoceros (*Ceratotherium simum*), elephant (*Loxodonta africana*) and African buffalo (*Syncerus caffer*)), as well as crocodile (*Crocodylus niloticus*) and hippopotamus (*Hippopotamus amphibius*).

Close encounters between ridden horses and game species are unnatural and potentially stressful situations, and horseback safaris may have adverse impacts on both the domestic horses being used to carry tourists and the wildlife they have come to observe. Horses and herbivorous plains game species typically use locomotion as their anti-predation strategy, meaning they are highly vigilant, sensitive to the behaviour of other individuals within their group and have a well-developed flight response [12,13,14,15]. Grazing ungulates are also known to respond to behavioural unease of other herbivorous prey species within their proximity [16,17]. The potential for two-way emotional contagion [18] between horses and game species during horseback safari rides may significantly impact the welfare of both the horses and the game species they encounter.

While the welfare of equines involved with the tourist industry is increasingly prioritised by customers and operators [19,20,21], there has yet to be any research published into the welfare implications of use as a horseback safari horse. Indeed, intrinsic welfare costs have not been investigated within many sectors of the equestrian tourism industry, although it has in other working horse roles, such as police horses [22], working equids in developing countries [23,24], racehorses and endurance horses [25]. Björlinger and Johansson [26] and Giampiccoli [27] investigated welfare standards in horse and mule tourism, respectively, identifying key issues such as poorly fitting tack, overwork, and limited access to water and good quality feed. However, neither evaluated the impact of the specific activity the horses and mules were being asked to perform.

The welfare consequences of human disturbance on plains game species has received less research attention than the more charismatic Big Five species. In impala in the Serengeti, faecal glucocorticoid metabolite (FGM) concentration is affected more by forage quality than human disturbance [28], and although impala may be more vigilant in tourist areas, there is some evidence of habituation to human presence [29]. Other research has reported diurnal changes in African ungulate behaviour as a result of human disturbance: in areas where human hunting is allowed, three large African ungulates (impala (*Aepyceros melampus*), greater kudu (*Tragelaphus strepsiceros*) and sable antelope (*Hippotragus niger*)) are more likely to drink at night [30], presumably due to hunting pressures. Diurnal and seasonal changes in grazing behaviour were observed by Schuette et al. [31] as ungulates selected a foraging strategy that was optimal for the level of human disturbance, the risk of predation and the vegetation quality. While Yamashita et al. [15] identified an influence of human settlements on the antipredator behaviour exhibited by African ungulates, they highlight the lack of detailed information on how different human activities may affect wildlife behaviour. The immediate, acute, impact of an encounter with tourists does not appear to have been considered previously.

Our primary hypothesis was that horseback safari rides would be stressful for both the horses and the plains game species they encounter, with a secondary hypothesis that that response will vary with game species. Therefore, this study aims to provide a preliminary insight into the behavioural responses of horses and plains game species as a proxy measure of the potential welfare implications of horseback safaris.

## 2. Materials and Methods

### 2.1. Ethics

Human and animal ethical approval for this study was granted by the following University of Bristol ethics committees: the Animal Welfare and Ethical Review Body (AWERB) and the Faculty of Health Science Student Research Ethics Committee (HSSREC; Ref: 65602). All riders participating in the study were provided with a Participant Information Sheet and gave informed consent to participate via reading and signing consent forms. Riders were assigned a Participant Identification Number to ensure anonymity.

### 2.2. Study Area

The study was conducted at an equestrian centre set in a popular private game reserve in Gweru, Zimbabwe. The reserve is 1214 hectares of savannah grassland and houses a variety of plains game species ranging freely including blue wildebeest (*Connochaetes taurinus*), impala (*Aepyceros melampus*), South African giraffe (*Giraffa camelopardalis giraffa*), Burchell’s zebra (*Equus quagga burchellii*), greater kudu (*Tragelaphus strepsiceros*), waterbuck (*Kobus ellipsiprymnus*) and red hartebeest (*Alcelaphus buselaphus caama*). All of the aforementioned species were observed during the study and are hereafter referred to collectively as plains game species for the purposes of this study.

It should be noted that captive lions and elephants also existed within the reserve. However, these species were both part of conservation projects and not free-ranging. Their enclosures were located in a completely separate area of the reserve away from the safari ride routes and horses. Therefore, neither species would have been directly encountered by the horses or game species, although they may have been aware of their presence through auditory and olfactory means. No hunting by humans was permitted within the reserve.

### 2.3. Horses

The centre was home to 30 horses at the time of the study which are used for a variety of disciplines including horseback safaris, where a guide takes guests around the park on horseback in search of game. The horses were either owned by the centre or kept on working livery and were all used regularly for horseback safari rides. However, due to illness and lameness, only 20 horses were involved in the study (see Table 1 for details of horses). Of these 20, 25% (n = 5) were safari horses, native African bush ponies which are bred and ideally suited for safari riding. The remaining 75% (n = 15) were Thoroughbred ex-racehorses. The Thoroughbreds were mainly used for polocrosse and show jumping but were also used for safari rides. Several had temperaments suitable for riding by beginners; however, many were only used by experienced riders. The horses’ management remained consistent for the duration of the study. All horses were fed a concentrate feed twice daily at 8:00 a.m. and 4:00 p.m. with appropriate quantities for their body condition and workload. The frequency and intensity of each horse’s exercise regime was also kept the same. All horses spent the day in small paddocks with grass and supplemented hay as it was dry season when they were not working. The Thoroughbred horses were kept individually stabled at night, each with a hay net, and the safari horses were allowed to roam the game reserve.

### 2.4. Riders

Horses were allocated to the riders by the safari organisers based on rider height, weight and self-reported riding experience. The researcher had no influence over horse allocation, or the specific horses used on each ride.

### 2.5. Data Collection

Prior to formal data collection, a week-long reconnaissance study was conducted for method refinement. Data collection was carried out on all rides occurring throughout June and July 2018. An encounter was defined as the interaction between at least one game animal (of the seven herbivorous plains game species described earlier) and the group of horses on the ride, starting and ending when the guide leading the ride began to purposefully approach or retreat from the game, respectively.

Due to the observational nature of the study, it was only possible to collect data when a ride was scheduled. The species of game encountered during each ride was purely due to chance.

Before each ride, the horse and rider combinations, time and weather conditions were recorded. The researcher then joined each ride on horseback, remaining at the rear of the ride for the best view. The researcher and their horse were included as a rider and subject, respectively. Video footage was recorded throughout the ride on a GoPro HERO (2018) Action Camera (GoPro Inc, California, USA), which was attached to the researcher’s riding hat via a head strap.

Although before the safari ride, riders had described their riding experience to the safari organisers to aid allocation of the most suitable horse, for study purposes their riding ability was scored by the researcher during the ride and each rider assigned a riding ability score from 1 (Total Beginner) to 7 (Professional) (see Table 2 for scores).

Following each ride, the video footage was analysed. For each encounter, the game species and number of individuals in the group were recorded, as well as the duration of the encounter, in minutes and seconds, from the start when the guide began to purposefully approach the game, and the end when the guide began to purposefully retreat from the game.

### 2.6. Game Behaviour

The response of the game to the approaching horses was recorded by continuous sampling using a predefined ethogram consisting of three categories of behaviour: stationary behaviour, approach behaviour and retreat behaviour with ordinal options defined within each category (Table 3). Behavioural scores were taken at the start and end of each encounter (initial score and final score), and the most extreme behaviour seen in each behavioural category during the encounter was also recorded (extreme score).

For encounters where there was more than one game animal present, one initial, one final and one extreme score was assigned to the whole group of that game species for the encounter, according to the mode, or most common behaviour displayed. This is because individuals within herds generally perform the same or similar behaviours as each other; this is the ‘mood’ of the herd [34].

**Table 3:** Ethogram of game response behaviours to encounters with horses on safari rides.

Behaviour	Measurement		Definition
Stationary	Scale		
	1	Lying down	Sternal or lateral recumbency [35]
	2	Grazing/Browsing	(As relevant for the species) Grazing: Standing with head down eating grassy vegetation. Vegetation is gathered and broken off with the lips and tongue [35,36] Browsing: Standing with head up eating from trees and shrubs. Vegetation is gathered and broken off with the lips and tongue [35].
	3	Standing at rest	Upright stance in a relaxed posture with head slightly lowered and eyes partly closed. Inactive and may be weight bearing on 3 legs [35].
	4	Standing alert	Upright stance with a rigid body position and neck elevated and head upright. Ears stiffly upright and pointing forwards. Focused eyes open and alert. Nostrils may be dilated [36,37].
Approach	Scale		
	1	Approach at walk	Forward movement towards the horses at a walk; a slow 4 beat gait [35,38].
	2	Approach at trot	Forward movement towards the horses at a trot; a 2 beat gait with diagonal pairs [35,38].
	3	Approach at canter	Forward movement towards the horses at a canter; a 3 beat medium speed gait [35,38].
	4	Approach at gallop	Forward movement towards the horses at a gallop; a 4 beat fast gait [35,38].
Retreat	1	Back up	Backward movement to maintain or increase distance from the horses, by reversing at a walk [35].
	2	Retreat at walk	Forward movement to maintain or increase distance from the horses at a walk; a slow 4 beat gait [35,36,38].
	3	Retreat at trot	Movement to maintain or increase distance from the horses at a trot; a 2 beat gait with diagonal pairs [35,36,38].
	4	Retreat at canter	Movement to maintain or increase distance from the horses at a canter; a 3 beat medium speed gait [35,36,38].
	5	Retreat at gallop	Movement to maintain or increase distance from the horses at a gallop; a 4 beat fast gait [35,36,38].

### 2.7. Horse Behaviour

As the video footage was recorded from the researcher’s position behind the ride during the encounters, behavioural scores could be taken for each individual horse using focal sampling and an ethogram (Table 4). The ethogram was comparable to that developed for the game species, with the addition of the category ‘ear position’ and shying, bucking and rearing behaviour. The footage was analysed continuously throughout the encounter. Three behavioural responses for each horse were recorded: at the start (initial score) for the first behaviour displayed, and end (final score) for the last behaviour displayed, as well as the most extreme score observed throughout the encounter for each category (extreme score). If a behaviour was not observed, e.g., a stationary score could not be given because the horse was showing an approach behaviour, that category was recorded as zero. There were also additional event behaviours (shy, rear and buck) in the equine ethogram which were recorded as frequencies.

While pilot testing indicated that reporting a group mood for game species using the behavioural mode adequately captured the response of the game to encounters with the horseback safari, the individual horses on a ride showed a wider range of behaviours in the same situation. Reporting the mode for each behavioural category failed to capture this variation. Consequently, behavioural scores were recorded for each horse at each encounter.

Distance between the horses and game was intended to be recorded, however following the pilot study this was excluded due to logistical difficulties in recording accurate distances between the multiple individuals in the groups of horses and game.

### 2.8. Data Analysis

All data were recorded in Microsoft Excel (Microsoft Office, Microsoft Corporation, Redmond, WA, USA), excluding pilot study data which were not included in the final analysis.

A descriptive account of the behavioural response of game and horses is reported as mean initial, final and extreme behavioural scores for each behaviour category across all encounters grouped by the seven plains game species observed.

As both horses and game species were in groups at the time of the encounters, the individual level data were not truly independent. This confounding effect meant that statistical analysis was inappropriate for these data. Consequently, only descriptive results are reported.

## 3. Results

In total, 17 group safari rides were observed over the data collection period. The rides lasted for a mean duration of two hours (range 1 h 14 min–2 h 22 min) and during these rides all 20 horses and 50 different riders were observed (the researcher, four full-time staff, 22 equestrian centre volunteer staff and 23 guests). Rider ability as scored by the researcher ranged from 1 (total beginner) to 7 (professional), with a median score of 4 corresponding to confident novice. Female riders made up 72% (n = 36) of the rider cohort. There were a median of 7 horses and riders per ride, with a range from 2–11.

There were 72 encounters with plains game species across the 17 rides, with a mean of 4.24 encounters per ride (range 1–8) and a mean encounter duration of 2 min 43 s (range 38 s to 7 min 32 s), generating 196 min of footage of horseback safari horses–plains game species encounters.

### 3.1. Response of Game to Horses

**Figure 1:**
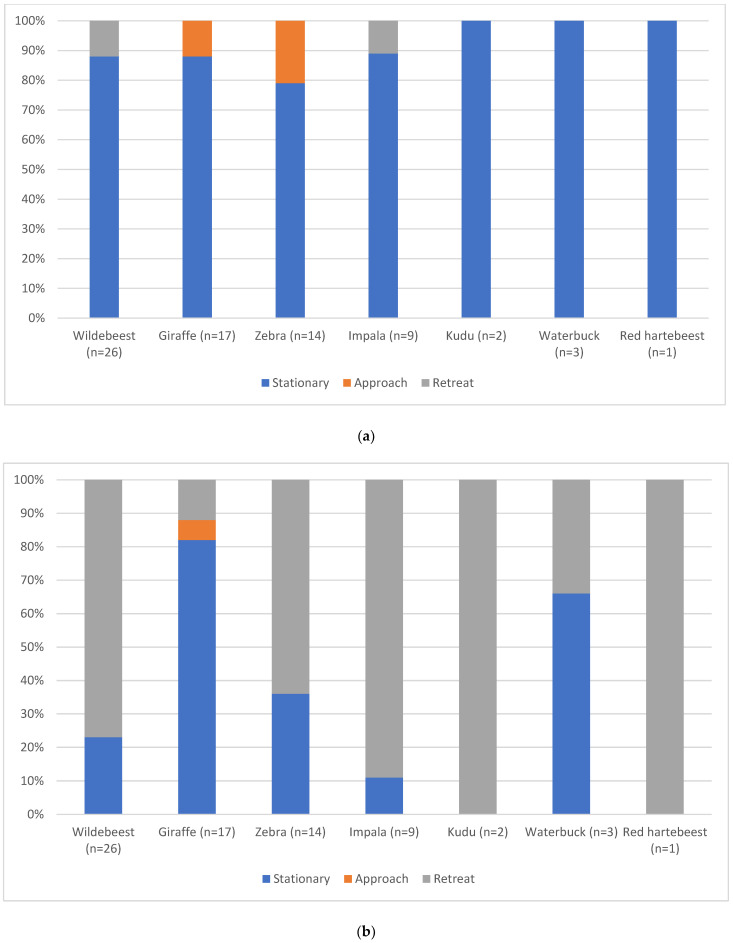
(**a**) Behavioural response of the seven plains game species observed towards the approach of the horseback safari ride at the start of encounters (percentage across all encounters). (**b**) Behavioural response of the seven plains game species observed at the end of encounters with the horseback safari ride (percentage across all encounters).

Wildebeest were the most frequently encountered plains game species (26/72 encounters; Table 5) and were also encountered in the biggest groups, while red hartebeest were the least frequently encountered with only one individual encountered across the 17 safari rides (1/72 encounters; Table 5). Response to the approaching horses varied between species (Figure 1a), with zebra approaching the horses at a walk in 21% of encounters, and retreat behaviours seen when horses approached the game in 12% of wildebeest and 11% of impala encounters. Most frequently, game responded to the approaching horses by standing still, either grazing/browsing, standing at rest or standing alert. Kudu and impala more frequently retreated from the horses at higher speeds (Figure 1b; Table 5), thereby ending encounters rather than the horses moving away first.

### 3.2. Response of Horses to Game

**Figure 2:**
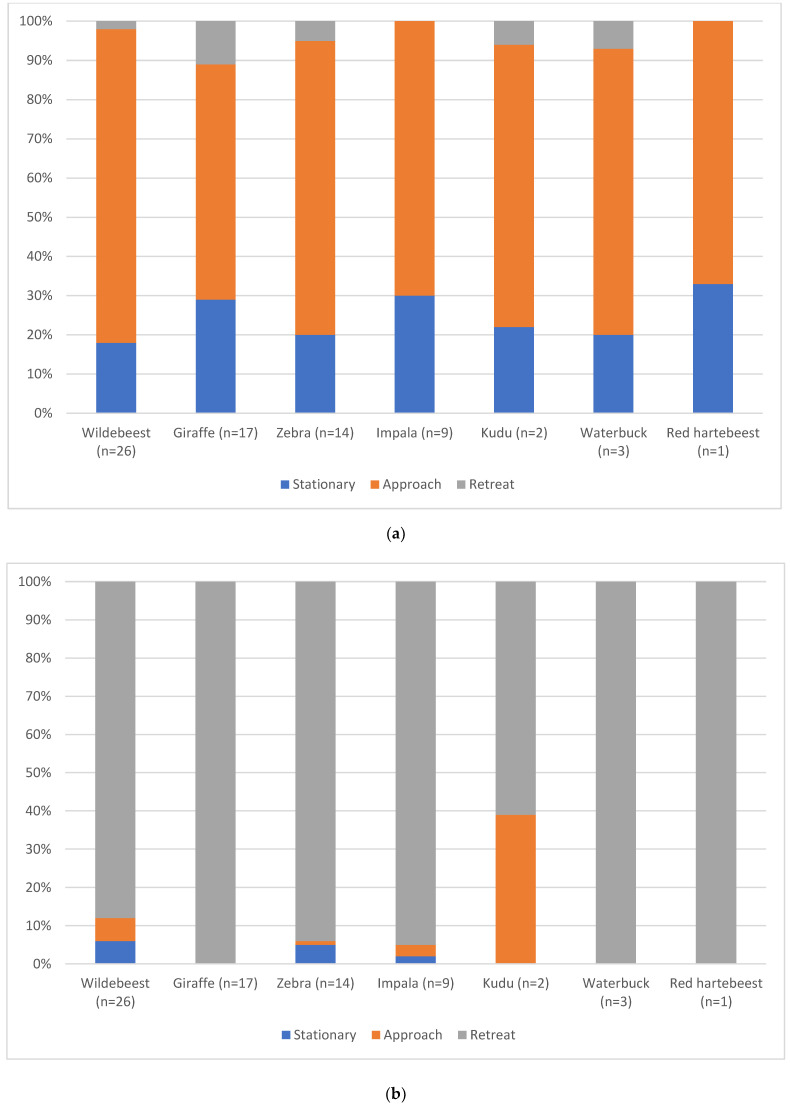
(**a**) Behavioural response of the horses in the horseback safari ride at the start of encounters with the seven plains game species observed (percentage across all encounters). (**b**) Behavioural response of the horses in the horseback safari ride at the end of encounters with the seven species of plains game species observed (percentage across all encounters).

The response of horses to game varied between horses and with the game species they were encountering (Table 6; Figure 2). Horses most frequently approached game in walk or trot, although horses in 11% of giraffe encounters were observed to retreat from the encounter by walking forward. The most extreme ear position was ears held back during encounters with wildebeest, giraffe and impala. Two horses retreated by cantering from the same encounter with zebra, and one from an encounter with wildebeest. Fourteen horses, across nine encounters, retreated from wildebeest at a trot, nine horses across six encounters retreated from zebra at a trot and nine horses across seven encounters retreated from giraffe at a trot. All encounters with giraffe, waterbuck and red hartebeest ended when the horses moved away at a walk, as did the majority of encounters with impala and zebra.

Across the 17 rides (72 encounters), there were 37 shies by twelve horses, with some individual horses exhibiting up to four instances of shying during one encounter. There were 8 shies during encounters with wildebeest (n = 6 horses), 14 during encounters with giraffe (n = 3 horses) 10 shies with zebra (n = 7 horses), 2 with impala (n = 2 horses), 2 with kudu (n = 1 horse), 1 with waterbuck (n = 1 horse). No horses shied during the sole encounter with Red Hartebeest. Of the twelve horses observed to shy, two were Safari horses and ten were Thoroughbreds.

No horses were observed to rear or buck during any of the encounters with plains game species on any of the rides.

## 4. Discussion

This preliminary study provides some initial insights into the welfare implications of horseback safari rides for both the horses and the plains game species they meet, based on the behaviour of both species at the time of the encounter.

The findings indicate that there were differences between game species in how they responded to their encounters with the horseback safari ride. Interestingly, zebra were typically calm and curious around the horses with 12% approaching the ride at the start of the encounter. The Burchell’s zebra (*Equus quagga burchellii*) encountered in this study are a subspecies of the plains zebra which are reported to be highly vigilant and reactive to human approach [36]. The findings from this study suggest that zebra may recognise horses as fellow equids and therefore not perceive them as a threat, despite the presence of human riders. Generally, the antelope species (wildebeest, impala, kudu, waterbuck and hartebeest) were highly alert and exhibited flight response behaviours, often retreating at speed. The response of kudu was the most extreme as 100% retreated at a gallop, although it should be noted that, due to the small sample sizes, conclusions about the behaviour of the kudu, waterbuck and hartebeest cannot reliably be made.

The likelihood of encountering each species of game can in part be explained by their prevalence in the park, but also corresponds with their behaviour. Wildebeest were the most commonly encountered, which was expected due to their high abundance. They were also found to be less vigilant and prone to flight than the other antelope species, which may be due to habituation or simply their natural behaviour [40]. Although there are far fewer giraffe and zebra in the park, they were often encountered. This may be explained by their behaviour as they were found to be the least vigilant and most curious animals; they were the most likely to approach and least likely to retreat from the ride. Despite the large numbers of impala in the park, they were not often encountered, and this may be due to their high vigilance and flighty, easily alarmed nature [34]. The response behaviours observed by impala also support Schenkel’s [34] findings of their general behaviour. Waterbuck, hartebeest and kudu were rarely seen which is likely due to low numbers in the reserve, in addition to their high vigilance. Some degree of habituation to horseback safaris by the plains game species encountered was likely, as has been suggested in other human encounters [15] and in other species visited by tourists [41], and this may also have affected the behaviour observed.

Equine response behaviour appeared to be influenced by the species of game encountered. The horses seemed more wary of giraffe than the other species, with a higher percentage of horses showing stationary and retreat behaviour at the start of giraffe encounters. They were also most likely to shy at the giraffe. Flight animals such as horses are more likely to flee from a persistent threat [34]. If the game retreats, it increases the distance between itself and the horses, thereby reducing the need for the horse to do so itself. However, the exhibited response behaviours towards zebra contradict this; the zebra showed low vigilance behaviour similar to the giraffe, and were observed to actively approach the horses, but this did not result in more flight responses from the horses. This may therefore support the earlier suggestion that zebra and horses recognise each other as equids and are consequently more at ease with one another. In addition to the influence of the game behaviour, it is possible that the differences in equine behavioural responses to different game species may also be affected by their size, appearance and smell.

Overall, most horses displayed vigilant response behaviours during game encounters, with ears pricked forwards and standing alert. These behaviours do not, however, necessarily indicate fear or stress, but may rather show alertness, interest and curiosity. Flight behaviour, such as shying or retreating, is the most extreme response [36] and was rarely observed except for the occasional shy or backing up (4% of encounters). The horses were generally willing to calmly approach the game at a walk (71% of encounters). These results suggest that the horses did not usually exhibit behavioural indicators of severe stress during game encounters.

Individual differences in how the horses responded to game encounters were evident but are impossible to draw out in this observational study. Personality differences may be involved [42] and are known to be associated with breed differences [43]. Within our sample of 20 horses, clear breed/type differences were observed. Thoroughbreds were more vigilant and less likely to approach the game than safari horses. Thoroughbred ex-racehorses are generally thought to be more highly strung [44] and are bred to be highly reactive for racing [43]. The Thoroughbreds in this study were generally younger and less experienced than their safari horse counterparts. This may have influenced the breed differences, as older horses with more experience of game encounters are more likely to be habituated to the situation and therefore be calmer and less reactive. Age is therefore a significant confounder that should be controlled for in further studies, alongside management. In this study the Thoroughbreds were stabled at night whereas the safari horses were grazed out in the game reserve. This meant that safari horses had greater exposure to game species. Furthermore, individual stabling has been shown to affect equine responsiveness to the environment [45]. It is important to note that behaviour may not necessarily reflect the internal stress state of the animal, particularly in prey species such as the horse, as it is adaptive for them to conceal their awareness and fear [36]. Additionally, horses are trained to suppress their natural reactions to perform behaviours desired by their riders [42]. The rider is likely to influence a horse’s behavioural response [46]; an experienced rider is more likely to give appropriate ridden cues (such as leg or rein aids) and ask for specific behaviours to control their horse, and the horse may be affected by the riders’ emotions and confidence [47]. Unfortunately, we were unable to explore this effect with data from the current study. The data do illustrate the potential value of employing gradual habituation protocols for new and young horses, to introduce them to all species of game they may encounter in a calm and positive manner.

There were a number of limitations to this study which impair interpretation of the findings. Observations were conducted opportunistically at one horseback safari establishment where the researcher was unable to control any of the variables such as horse age, breed, management, rider ability and game species encountered. Furthermore, both the horses and game species were with at least one other conspecific during each encounter (with the exceptions of the waterbuck and red hartebeest), so the behaviour of each individual within the group was potentially influenced by the behaviour of other conspecifics, as well as by the other species (game or horses). This limited the ability to conduct any meaningful statistical analysis on the data as it was impossible to control for all confounders nor to treat the data as independent. Physiological measures, such as faecal glucocorticoid metabolites, heart rate and heart rate variability, were not taken to support the behavioural observations, although as previously noted in many instances behavioural and physiological indicators of stress do not always align [22,42,48]. Future studies should attempt to tease out these confounding variables to produce a more robust evaluation of the welfare implications of safari rides on the animals involved, including evaluating the influences of variables such as horse breed/type and rider ability.

That said, our findings still have merit and suggest that the welfare implications of horseback safari encounters are generally not extreme for either party. In a recent review, Bateman and Fleming [49] conclude that there is often little empirical evidence for negative interpretations of wildlife–tourist encounters. Wildlife–tourist encounters may impact the animals involved in a myriad of ways both short- and long-term, directly and indirectly [49]. This study looked at the short-term behavioural impact of direct encounters for the horses and game species involved to provide an initial insight into the potential implications of this industry. Future studies would benefit from exploring the longer-term implications of these encounters using a range of behavioural, physiological and survival measures.

## 5. Animal Welfare Implications and Conclusions

This study aimed to provide an initial insight into the behavioural response of horses and plains game species during horseback safari encounters, an area which has not been previously studied. The behavioural responses exhibited by the horses and game animals suggest that game encounters can elicit a stress response in both animal groups, although it is not usually extreme, indicating that some degree of habituation has potentially occurred. These preliminary findings imply that the welfare implications of horseback safari rides are not entirely negative, although this varies with the game species encountered. Kudu, waterbuck and red hartebeest showed the most extreme flight response towards the ride, while giraffe and zebra appeared less affected, and even curious, with a small percentage approaching the horses. That said, the horses’ behaviour suggested that they were more wary of giraffe than the other game species. Balancing the welfare of both the horses and the plains game species along with the preferences of the tourists may be challenging in this context. Before firm conclusions can be drawn on the welfare implications of horseback safari rides, it is recommended that future studies utilise multiple welfare indicators, encompassing behavioural and physiological measures, and evaluate the long-term and indirect consequences of horseback safari rides. This information may then be used to inform best practice guidelines within the industry, such as maximum distances that different species can be approached by riders on horseback, to reduce stress, improve welfare, and ensure safety and enjoyment for riders.

## Figures and Tables

**Table 1:** Demographic information on the 20 horses used for the seventeen horseback safari rides observed.

Name	Sex	Age Category	Horse Type	Body Condition Score [32]	Minimum Rider Ability [33]
1	Mare	over 15	Safari Horse	3/9	Total Beginner
2	Gelding	6 to 10	Safari Horse	7/9	Total Beginner
3	Mare	over 15	Safari Horse	3/9	Total Beginner
4	Mare	over 15	Safari Horse	4/9	Total Beginner
5	Gelding	over 15	Safari Horse	4/9	Advanced Beginner
6	Mare	under 6	Thoroughbred	4/9	Confident Beginner
7	Gelding	under 6	Thoroughbred	4/9	Total Beginner
8	Gelding	6 to 10	Thoroughbred	5/9	Novice
9	Gelding	under 6	Thoroughbred	5/9	Total Beginner
10	Gelding	under 6	Thoroughbred	4/9	Advanced
11	Gelding	6 to 10	Thoroughbred	5/9	Confident Beginner
12	Gelding	under 6	Thoroughbred	5/9	Advanced Beginner
13	Gelding	under 6	Thoroughbred	4/9	Confident Beginner
14	Gelding	under 6	Thoroughbred	4/9	Confident Beginner
15	Mare	6 to 10	Thoroughbred	4/9	Intermediate
16	Mare	under 6	Thoroughbred	5/9	Confident Beginner
17	Mare	6 to 10	Thoroughbred	6/9	Novice
18	Mare	6 to 10	Thoroughbred	5/9	Intermediate
19	Gelding	6 to 10	Thoroughbred	4/9	Advanced
20	Gelding	11 to 15	Thoroughbred	5/9	Intermediate

**Table 2:** Riding ability scores used to categorise the riders that went on the seventeen horseback safari rides observed.

Score	Riding Ability	Description [33]
1	Total Beginner	Little if any experience with horses in general. Does not know general horse handling or basic commands to make a horse move, stop or turn.
2	Advanced Beginner	Little experience with a horse. Can mount and ask a horse to move, stop and turn unassisted. May be able to trot on a well-schooled horse.
3	Confident Beginner	Knowledge of an advanced beginner plus the ability to handle a more difficult horse; they are confident to use more persuasive aids. May be able to rise to the trot.
4	Novice	Pretty basic experience with horses. Can catch and tack up a horse and mount unassisted. They can change direction, circle and complete upward and downward transitions. They can trot and canter on a well-schooled horse.
5	Intermediate	Secure in the saddle (secure seat), can rise to the trot and know their diagonals and leads. Capable of riding a less experienced horse. Able to train to a more advanced level with a trainer’s assistance and may compete. Knowledgeable about horse care, breeds and disciplines.
6	Advanced	Able to ride most horses including working with and training young horses. Can complete advanced manoeuvres in their preferred disciplines and have competed successfully. Very knowledgeable about horse care, breeds and disciplines.
7	Professional	Paid to ride horses and can break, train and handle problem horses. Able to teach both horse and rider and have competed at a high level.

**Table 4:** Ethogram of equine response behaviours to encounters with game species.

Behaviour	Measurement		Definition
Shy	Frequency		Sudden veering to avoid novel or fear-provoking stimuli [35].
Rear	Frequency		Both forelegs raised into the air therefore weight bearing on the hindquarters [35,39].
Buck	Frequency		Both hindlegs lifted off the ground with backward extension and weight shifted onto the forelegs [35,39].
Ear Position	Scale		
	1	Rest	Ears are relaxed and lateral or gently back and stationary for 3+ s [35,39].
	2	Scanning	Ears moving back and forth at varying speeds [39].
	3	One ear on rider	One ear focused on rider and the other pricked up pointing forwards and stationary for ≥3 s [39].
	4	Forward	Ears pricked up pointing forwards and stationary for 3+ s [39].
	5	Back	Ears pointing caudally for 3+ s [39].
	6	Flat back	Ears pressed caudally flat against head and neck [39].
Stationary	Scale		
	1	Submissive posture	Standing quiet with head lowered. Unresponsive to stimuli until given a command by rider [35].
	2	Grazing	Standing with head down eating grassy vegetation. Vegetation is gathered and broken off with the lips and tongue [35].
	3	Standing at rest	Upright stance in a relaxed posture with head slightly lowered and eyes may be partly closed. Inactive and may be weight bearing on 3 legs [35].
	4	Standing alert	Upright stance with a rigid body position and neck elevated and head upright. Ears stiffly upright and pointing forwards. Focused eyes open and alert. Nostrils may be dilated [37].
Approach	Scale		
	1	Approach at walk	Forward movement towards the game at a walk; a slow 4 beat gait [35].
	2	Approach at trot	Forward movement towards the game at a trot; a 2 beat gait with diagonal pairs [35].
	3	Approach at canter	Forward movement towards the game at a canter; a 3 beat medium speed gait [35].
	4	Approach at gallop	Forward movement towards the game at a gallop; a 4 beat fast gait [35].
Retreat	Scale		
	1	Back up	Movement to maintain or increase distance from the game, by reversing at a walk [35].
	2	Retreat at walk	Movement to maintain or increase distance from the game at a forwards walk; a slow 4 beat gait [35].
	3	Retreat at trot	Movement to maintain or increase distance from the game at a trot; a 2 beat gait with diagonal pairs [35].
	4	Retreat at canter	Movement to maintain or increase distance from the game at a canter; a 3 beat medium speed gait [35].
	5	Retreat at gallop	Movement to maintain or increase distance from the game at a gallop; a 4 beat fast gait [35].

**Table 5:** Mean (range) of the behavioural response observed by each of the seven species of plains game encountered towards the horses on the seventeen safari rides observed in the study as scored using the ethogram described in Table 3. Initial behaviour refers to the behaviour observed at the start of the encounter, final behaviour was the behaviour observed at the end of the encounter. Extreme behaviour was the most extreme behaviour observed in each of the three behaviour categories (stationary, approach and retreat).

	N Encounters /Mean Encounter Duration (Range in Secs)	Mean Game Species Group Size (Range)	Behaviour	Mean Initial Behaviour (Range)	Mean Final Behaviour (Range)	Mean Extreme Behaviour (Range)
Wildebeest	26 2:35 (0:38–5:42)	15 (1–29)	Stationary Approach Retreat	2.22 (1–4) - 2.00 (-)	3.83 (3–4) - 2.75 (2–4)	3.92 (2–4) 1.40 (1–3) 3.15 (2–4)
Giraffe	17 3:18 (1:22–5:13)	2 (1–5)	Stationary Approach Retreat	3.00 (2–4) 1.00 (-) -	3.42 (2–4) 1.00 (-) 2.00 (-)	3.94 (3–4) 1.00 (-) 2.00 (-)
Zebra	14 2:55 (0:43–7:32)	5 (2–8)	Stationary Approach Retreat	2.36 (1–4) 1.00 (-) -	4.00 (-) - 2.11 (2–3)	4.00 (-) 1.29 (1–3) 2.30 (2–4)
Impala	9 1:52 (0:43–3:37)	17 (9–32)	Stationary Approach Retreat	4.00 (-) - 1.00 (-)	4.00 (-) - 3.88 (2–5)	4.00 (-) 0.00 4.13 (4–5)
Kudu	2 2:17 (0:51–3:42)	5 (2–7)	Stationary Approach Retreat	4.00 (-) - -	- - 5.00 (-)	4.00 (-) 0.00 5.00 (-)
Waterbuck	3 2:41 (1:22–3:27)	1 (1)	Stationary Approach Retreat	3.00 (1–4) - -	2.50 (1–4) - 2.00 (-)	3.00 (1–4) 1.00 (-) 2.00 (-)
Red Hartebeest	1 1:50 (NA)	1 (NA)	Stationary Approach Retreat	4.00 (-) - -	- - 2.00 (-)	4.00 (-) 0.00 3.00 (-)

N/A = not applicable.

**Table 6:** Mean (range) of the behavioural response observed for horseback safari horses towards each of the seven species of plains game encountered on the seventeen safari rides observed in the study as scored using the ethogram described in Table 4. Initial behaviour refers to the behaviour observed at the start of the encounter, final behaviour was the behaviour observed at the end of the encounter. Extreme behaviour was the most extreme behaviour observed in each of the three behaviour categories (stationary, approach and retreat).

	Behaviour	Mean Initial Behaviour	Mean Final Behaviour	Mean Extreme Behaviour
Wildebeest	Stationary Approach Retreat EP ^*^	3.80 (2–4) 1.04 (1–2) 1.00 (-) 2.96 (1–5) 1 ear on rider	3.80 (3–4) 1.00 (-) 2.01 (1–3) 3.02 (1–5) 1 ear on rider	4.00 (2–4) 2.00 (1–2) 4.00 (1–4) 3.80 (1–5) Forward
Giraffe	Stationary Approach Retreat EP ^*^	3.67 (2–4) 1.00 (-) 1.00 (-) 2.97 (1–5) 1 ear on rider	- - 2.02 (2–3) 3.04 (1–4) 1 ear on rider	4.00 (2–4) 2.00 (1–2) 3.00 (1–3) 3.77 (1–5) Forward
Zebra	Stationary Approach Retreat EP ^*^	3.63 (2–4) 1.00 (-) 1.00 (-) 2.92 (1–5) 1 ear on rider	4.00 (-) 1.00 (-) 2.01 (2–3) 3.12 (1–5) 1 ear on rider	4.00 (2–4) 2.00 (1–2) 3.00 (2–3) 3.75 (1–5) Forward
Impala	Stationary Approach Retreat EP ^*^	3.90 (3–4) 1.02 (1–2) - 2.92 (1–5) 1 ear on rider	4.00 (-) 1.00 (-) 2.00 (-) 2.93 (1–5) 1 ear on rider	4.00 (2–4) 2.00 (1–2) 3.00 (2–3) 3.72 (1–5) Forward
Kudu	Stationary Approach Retreat EP ^*^	3.50 (3–4) 1.08 (1–2) 1.00 (-) 3.17 (1–4) 1 ear on rider	- 1.00 (-) 1.91 (1–2) 2.94 (1–4) 1 ear on rider	4.00 (3–4) 1.00 (-) 2.00 (-) 3.83 (2–4) Forward
Waterbuck	Stationary Approach Retreat EP ^*^	4.00 (-) 1.00 (-) 1.00 (-) 3.27 (1–4) 1 ear on rider	- - 2.00 (-) 3.07 (1–4) 1 ear on rider	4.00 (3–4) 1.00 (-) 2.00 (-) 3.53 (2–4) Forward
Red Hartebeest	Stationary Approach Retreat EP ^*^	3.5 (3–4) 1.00 (-) - 2.33 (1–4) Scanning	- - 2.00 (-) 3.00 (1–4) 1 ear on rider	4.00 (3–4) 1.00 (-) 2.00 (-) 3.83 (3–4) Forward

EP ^*^ = ear position.

## Data Availability

The data presented in this study are available on request from the corresponding author. The data are not publicly available as this was not explicitly consented to by study participants.

## References

[1] Buckley R., Mossaz A. (2018). Private conservation funding from wildlife tourism enterprises in sub-Saharan Africa: Conservation marketing beliefs and practices. Biol. Conserv..

[2] Shannon G., Larson C.L., Reed S.E., Crooks K.R., Angeloni L.M., Blumstein D., Geffroy B., Samia D., Bessa E. (2017). Ecological Consequences of Ecotourism for Wildlife Populations and Communities. Ecotourism’s Promise and Peril.

[3] Krüger O. (2005). The role of ecotourism in conservation: Panacea or Pandora’s box?. Biodivers. Conserv..

[4] Shutt K., Heistermann M., Kasim A., Todd A., Kalousova B., Profosouva I., Petzelkova K., Fuh T., Dicky J.-F., Bopalanzognako J.-B. (2014). Effects of habituation, research and ecotourism on faecal glucocorti-coid metabolites in wild western lowland gorillas: Implications for conservation management. Biol. Conserv..

[5] Zhou Y., Buesching C.D., Newman C., Kaneko Y., Xie Z., Macdonald D.W. (2013). Balancing the benefits of eco-tourism and development: The effects of visitor trail-use on mammals in a Protected Area in rapidly develop-ing China. Biol. Conserv..

[6] Bhandari M. (2014). Is Tourism Always Beneficial? A Case Study from Masai Mara National Reserve, Narok, Kenya. Pac. J. Sci. Technol..

[7] Szott I.D., Pretorius Y., Ganswindt A., Koyama N.F. (2020). Physiological stress response of African elephants to wildlife tourism in Madikwe Game Reserve, South Africa. Wildl. Res..

[8] Behie A.M., Pavelka M.S., Chapman C.A. (2010). Sources of variation in fecal cortisol levels in howler monkeys in belize. Am. J. Primatol..

[9] Thiel D., Jenni-Eiermann S., Braunisch V., Palme R., Jenni L. (2007). Ski tourism affects habitat use and evokes a physiological stress response in capercaillie *Tetrao urogallus*: A new methodological approach. J. Appl. Ecol..

[10] Rehnus M., Wehrle M., Palme R. (2013). Mountain haresLepus timidusand tourism: Stress events and reactions. J. Appl. Ecol..

[11] Ranaweerage E., Ranjeewa A.D., Sugimoto K. (2015). Tourism-induced disturbance of wildlife in protected areas: A case study of free ranging elephants in Sri Lanka. Glob. Ecol. Conserv..

[12] McGreevy P. (2004). Equine Behavior: A Guide for Veterinarians and Equine Scientists.

[13] Simpson H.I., Rands S.A., Nicol C.J. (2012). Social structure, vigilance and behaviour of plains zebra (Equus burchellii): A 5-year case study of individuals living on a managed wildlife reserve. Acta Thériol..

[14] Tarakini T., Crosmary W.-G., Fritz H., Mundy P. (2014). Flight Behavioural Responses to Sport Hunting by Two African Herbivores. South Afr. J. Wildl. Res..

[15] Yamashita T., Gaynor K.M., Kioko J., Brashares J., Kiffner C. (2018). Antipredator behaviour of African ungulates around human settlements. Afr. J. Ecol..

[16] Schmitt M.H., Stears K., Wilmers C.C., Shrader A.M. (2014). Determining the relative importance of dilution and detection for zebra foraging in mixed-species herds. Anim. Behav..

[17] Schmitt M.H., Stears K., Shrader A.M. (2016). Zebra reduce predation risk in mixed-species herds by eavesdrop-ping on cues from giraffe. Behav. Ecol..

[18] Nakahashi W., Ohtsuki H. (2018). Evolution of emotional contagion in group-living animals. J. Theor. Biol..

[19] Helgadóttir G., Sigurðardóttir I. (2008). Horse-based Tourism: Community, Quality and Disinterest in Eco-nomic Value. Scand. J. Hosp. Tour..

[20] Fennell D.A. (2013). Tourism and Animal Welfare. Tour. Recreat. Res..

[21] Notzke C. (2019). Equestrian tourism: Animal agency observed. Curr. Issues Tour..

[22] Munsters C., Visser K., Broek J.V.D., Van Oldruitenborgh-Oosterbaan M.S. (2013). Quantifying stress in experienced and inexperienced mounted police horses, using heart rate, heart rate variability, behavior score and suitability score. J. Vet. Behav..

[23] Pritchard J., Lindberg A., Main D., Whay H. (2005). Assessment of the welfare of working horses, mules and donkeys, using health and behaviour parameters. Prev. Veter. Med..

[24] Burn C.C., Dennison T.L., Whay H.R. (2010). Environmental and demographic risk factors for poor welfare in working horses, donkeys and mules in developing countries. Veter. J..

[25] Witkowska-Piłaszewicz O., Grzędzicka J., Seń J., Czopowicz M., Żmigrodzka M., Winnicka A., Cywińska A., Carter C. (2021). Stress response after race and endurance training sessions and competitions in Arabian horses. Prev. Veter. Med..

[26] Björlinger K., Johansson S. (2016). Equestrian Tourism in Trinidad. Undergraduate Thesis.

[27] Giampiccoli A. (2017). The effect of the use of mules in tourism: A historical perspective. Afr. J. Hosp. Tour. Leis..

[28] Hunninck L., May R., Jackson C.R., Palme R., Røskaft E., Sheriff M.J. (2020). Consequences of climate-induced vegetation changes exceed those of human disturbance for wild impala in the Serengeti ecosystem. Conserv. Physiol..

[29] Muposhi V.K., Muvengwi J., Mandima L. (2016). Vigilance and foraging behaviour of impala (*Aepyceros melampus*) in an isolated and insularised ecosystem, Mukuvisi Woodlands, Zimbabwe. Ann. Soc. Behav. Sci..

[30] Crosmary W.-G., Valeix M., Fritz H., Madzikanda H., Côté S.D. (2012). African ungulates and their drinking problems: Hunting and predation risks constrain access to water. Anim. Behav..

[31] Schuette P., Creel S., Christianson D. (2016). Ungulate distributions in a rangeland with competitors, predators and pastoralists. J. Appl. Ecol..

[32] Henneke D.R., Potter G.D., Kreider J.L., Yeates B.F. (1983). Relationship between condition score, physical measurements and body fat percentage in mares. Equine Vet. J..

[33] The Long Riders Guild Academic Foundation 2014 Different Levels of Riding Ability. http://www.lrgaf.org/guide/ability.htm.

[34] Schenkel R. (1966). On sociology and behaviour in impala (*Aepyceros melampus* Lichenstein). Afr. J. Ecol..

[35] McDonnell S. (2003). A Practical Field Guide to Horse Behaviour: The Equid Ethogram.

[36] Brubaker A.S., Coss R.G. (2016). Effects of Single- and Mixed-Species Group Composition on the Flight Initiation Distances of Plains and Grevy’s Zebras. Int. J. Behav. Biol..

[37] Goodwin D. (2010). The importance of ethology in understanding the behaviour of the horse. Equine Vet. J..

[38] Van Klink L. (2016). Ungulate Response Behaviour to Tourist Activities in a South African Private Game Reserve. Master’s Thesis.

[39] Young T., Creighton E., Smith T., Hosie C. (2012). A novel scale of behavioural indicators of stress for use with domestic horses. Appl. Anim. Behav. Sci..

[40] Hunter L.T.B., Skinner J.D. (1998). Vigilance Behaviour in African Ungulates: The Role of Predation Pressure. Behaviour.

[41] Knight J. (2009). Making Wildlife Viewable: Habituation and Attraction. Soc. Anim..

[42] Squibb K., Griffin K., Favier R., Ijichi C. (2018). Poker Face: Discrepancies in behaviour and affective states in horses during stressful handing procedures. Appl. Anim. Behav. Sci..

[43] Lloyd A., Martin J., Bornett-Gauci H., Wilkinson R. (2007). Horse personality: Variation between breeds. Appl. Anim. Behav. Sci..

[44] Freestone J.F., Wolfsheimer K.J., Kamerling S.G., Church G., Hamra J., Bagwell C. (1991). Exercise induced hormonal and metabolic changes in Thoroughbred horses: Effects of conditioning and acepromazine. Equine Vet. J..

[45] Ruet A., LeMarchand J., Parias C., Mach N., Moisan M.-P., Foury A., Briant C., Lansade L. (2019). Housing Horses in Individual Boxes Is a Challenge with Regard to Welfare. Animals.

[46] Christensen J.W., Munk R., Hawson L., Palme R., Larsen T., Egenvall A., von Borstel U.U.K., Rørvang M.V. (2021). Rider effects on horses’ conflict behaviour, rein tension, physiological measures and rideability scores. Appl. Anim. Behav. Sci..

[47] Keeling L.J., Jonare L., Lanneborn L. (2009). Investigating horse–human interactions: The effect of a nervous human. Veter. J..

[48] Yarnell K., Hall C., Billett E. (2013). An assessment of the aversive nature of an animal management procedure (clipping) using behavioral and physiological measures. Physiol. Behav..

[49] Bateman P., Fleming P.A. (2017). Are negative effects of tourist activities on wildlife over-reported? A review of assessment methods and empirical results. Biol. Conserv..

